# Associations between meteorology and COVID-19 in early studies: Inconsistencies, uncertainties, and recommendations

**DOI:** 10.1016/j.onehlt.2021.100225

**Published:** 2021-02-09

**Authors:** Gaige Hunter Kerr, Hamada S. Badr, Lauren M. Gardner, Javier Perez-Saez, Benjamin F. Zaitchik

**Affiliations:** a Department of Occupational and Environmental Health, George Washington University, Washington, DC, USA; b Department of Earth and Planetary Sciences, Johns Hopkins University, Baltimore, MD, USA; c Department of Civil and Systems Engineering, Johns Hopkins University, Baltimore, MD, USA; d Bloomberg School of Public Health, Johns Hopkins University, Baltimore, MD, USA

**Keywords:** COVID-19, SARS-CoV-2, Temperature, Humidity, Meteorology, Virus transmission

## Abstract

Meteorological variables, such as the ambient temperature and humidity, play a well-established role in the seasonal transmission of respiratory viruses and influenza in temperate climates. Since the onset of the novel coronavirus disease 2019 (COVID-19) pandemic, a growing body of literature has attempted to characterize the sensitivity of COVID-19 to meteorological factors and thus understand how changes in the weather and seasonality may impede COVID-19 transmission. Here we select a subset of this literature, summarize the diversity in these studies' scopes and methodologies, and show the lack of consensus in their conclusions on the roles of temperature, humidity, and other meteorological factors on COVID-19 transmission dynamics. We discuss how several aspects of studies' methodologies may challenge direct comparisons across studies and inflate the importance of meteorological factors on COVID-19 transmission. We further comment on outstanding challenges for this area of research and how future studies might overcome them by carefully considering robust modeling approaches, adjusting for mediating and covariate effects, and choosing appropriate scales of analysis.

## Highlights

• Meteorology has contradicting roles in COVID-19 transmission in recent studies.
• Inconsistent adjustments for lag and mediation complicates interstudy comparison.
• We provide recommendations to avoid potentially spurious conclusions in future studies.

## 1 Introduction

The link of respiratory viruses and influenza with temperature and humidity in temperate climates is well established in the literature. During the pandemic of the novel coronavirus disease 2019 (COVID-19) caused by severe acute respiratory syndrome coronavirus 2 (SARS-CoV-2), questions have been raised about the meteorological sensitivity of COVID-19 transmission, and the scientific community has made concerted efforts to address these questions. Temperature and humidity impact respiratory diseases via their effect on host susceptibility, virus survival on fomites, the aerosolization of virus droplets, and human behavior [[1], [2], [3], [4], [5], [6], [7], [8]]. Other meteorological variables such as precipitation may also impact virus transmission outside of temperate regions [7].

While meteorological impacts on the transmission of COVID-19 might be plausibly hypothesized from the literature, statements touting the environmental sensitivity of COVID-19 may incur profound impacts and costs [9]. For example, policymakers eager to reopen economic activity may decide that a particular study justifies lifting social distancing and stay-at-home mandates because weather conditions are becoming unfavorable for COVID-19 transmission. It is thus imperative to understand what evidence supports these claims. *If* heat, humidity, and other meteorological variables have been shown to impact COVID-19 transmission in recent studies, how generalizable are these results given the limited data record and heterogeneities in human behavior, non-pharmaceutical interventions, and other confounding factors?

To understand the science behind these claims, we turn to the growing body of literature examining meteorological impacts on COVID-19 transmission. A flurry of preprint and published studies have been released since the onset of the pandemic. Of these, we select a subset of manuscripts that are representative of different approaches researchers have taken to study the sensitivity of COVID-19 to meteorology and draw recommendations for future studies and offer guidance on avoiding pitfalls that have confounded many early studies on this topic.

## 2 Methods

Preprint and published studies investigating the meteorological sensitivity of COVID-19 appear nearly daily, and the landscape of the literature is constantly changing. As such, the goal of this review is not to provide a systematic review of all studies but rather to holistically summarize key results and identify key decisions on study design, methodology, and data that can lead to divergent, and often unreliable, results. We also give recommendations for future work on this topic.

We use electronic databases (i.e., PubMed, Google Scholar) to search for manuscripts and thereafter manually check references to find other relevant studies. As our review is not intended to be exhaustive, we consider 43 studies, 23 of which are published and 20 of which appear as preprints. Although we acknowledge the potential for selection bias, the selected studies capture a wide range of methods, data sources, and geographic and temporal scopes. Moreover, the lack of consensus on the sensitivity of COVID-19 to meteorological factors in our selected studies is similar to reviews that have taken a comprehensive approach when selecting and summarizing existing studies [[10], [11], [12]].

## 3 Results from early studies

**Fig. 1:**
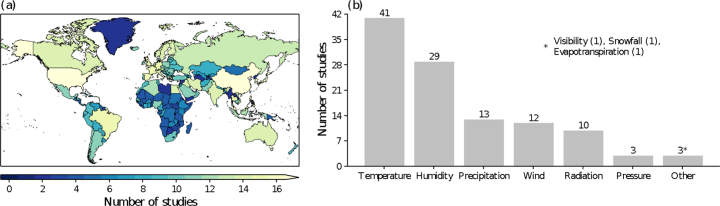
(a) Countries from which data were drawn by the 43 studies synthesized in this review. If one city was the focus of a study, the entire country was shaded. (b) Meteorological predictor variables considered by studies.

Recent preprint and peer-reviewed manuscripts on the meteorological sensitivity of COVID-19 generally limit their geographic scope to data-rich countries affected early in the pandemic such as China, Japan the U.S., and nations in Western Europe (Fig. 1a). Of the subset of articles covered by this review, China, the U.S., and Japan have the most studies examining the role of meteorological variables on COVID-19 within their borders, with 23, 20, and 16 studies drawing data from these countries, respectively. While Fig. 1a indicates that nations in the Global South have featured in studies, the studies focusing on this region generally employ global meteorological reanalyses and global case report data rather than homing in on specific countries or regions.

Nearly all studies featured in this review examine the role of temperature on COVID-19, and a majority of studies also consider humidity, either as a single independent variable or in tandem with other explanatory variables (Fig. 1b). Different expressions of temperature (e.g., minimum, maximum, diurnal range) and humidity (e.g., relative, specific, absolute) are used. The other meteorological variables considered in some studies (e.g., precipitation, UV radiation, wind) typically are included alongside temperature and humidity within their study designs.

The prominent use of temperature and humidity in our featured studies is supported by the past work on the seasonality of respiratory viruses noted in Section 1. Studies tend to use observed meteorological data from local agencies within particular countries (e.g., National Oceanic and Atmospheric Administration, Japan Meteorological Agency, Meteorological Department of Republic of Indonesia) [[13], [14], [15]] or from commercial weather services (e.g., Accuweather, WeatherUnderground) [16,17]. As was previously mentioned, studies with a global focus commonly exploit meteorological reanalyses [18,19].

We next explore whether these early studies reach similar conclusions regarding the role of meteorological factors on COVID-19. As temperature and humidity have an established role in virus transmission [e.g., [4], [5], [6]] and are most commonly examined in our featured studies (Fig. 1b), we focus on the subset of studies that have assessed the impact of these variables over the global (or quasi-global) domain. Since no location has undergone a complete annual cycle at this point in time and studies employ a “space-for-time substitution” [20], one might reasonably expect that the global domain would provide the most expansive range of meteorological conditions upon which to base conclusions.

**Fig. 2:**
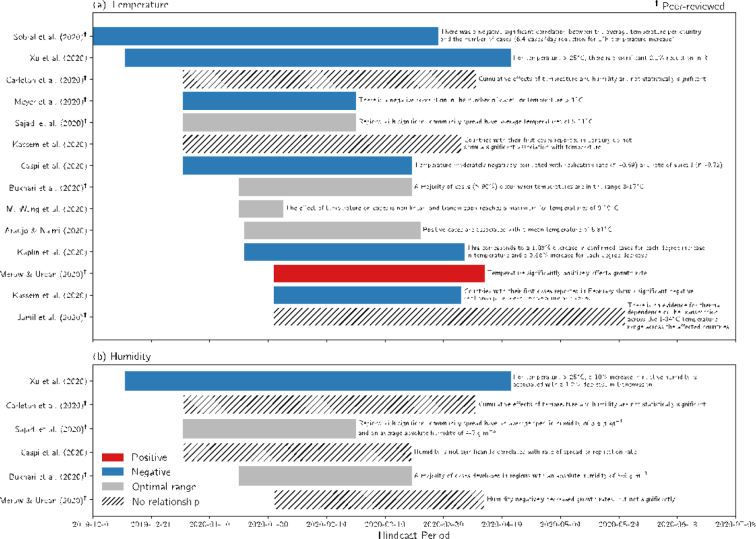
Studies focusing on the sensitivity of COVID-19 to (a) temperature and (b) humidity over the global domain versus their hindcast period. Studies are shaded by the sign or result of their key finding, and a brief summary of the study is provided. A positive relationship implies that increasing temperature or humidity is associated with additional COVID-19 cases or other COVID-19 transmission metrics, while the opposite is true for a negative relationship. Several studies do not provide directionality but rather report an optimal range.

Based on past work regarding the role of temperature on virus transmission, a negative relationship between COVID-19 and temperature is expected. This outcome, however, is not reflected in many global studies examining the effect of temperature on COVID-19 (Fig. 2a). Less than half of global studies find a negative relationship, while several of the other studies only report an optimal range for transmission [[21], [22], [23], [24]]. These optimal ranges span a wide distribution of temperatures (~0–17 °C), which may not be strictly unique to the winter season. The directionality of temperature-COVID-19 studies is also not a function of each study's hindcast period or length (Fig. 2a). Fewer studies have concentrated on the role of humidity on COVID-19 over the global domain compared with temperature but have similarly found varied results (Fig. 2b).

Unconsidered mediating or covariate effects associated with both the weather and transmission dynamics could change the direction or magnitude of results and complicate causal inference [25,26]. Potential mediators and covariates between COVID-19 and meteorological factors can be grouped into four key families: (1) demography (e.g., socioeconomic characteristics, population density), (2) policy (e.g., social distancing measures, surveillance and contact tracing), (3) human behavior (e.g., adherence to policy, travel patterns), and (4) epidemiology (e.g., herd immunity, seasonality in immune function, comorbidities).

**Fig. 3:**
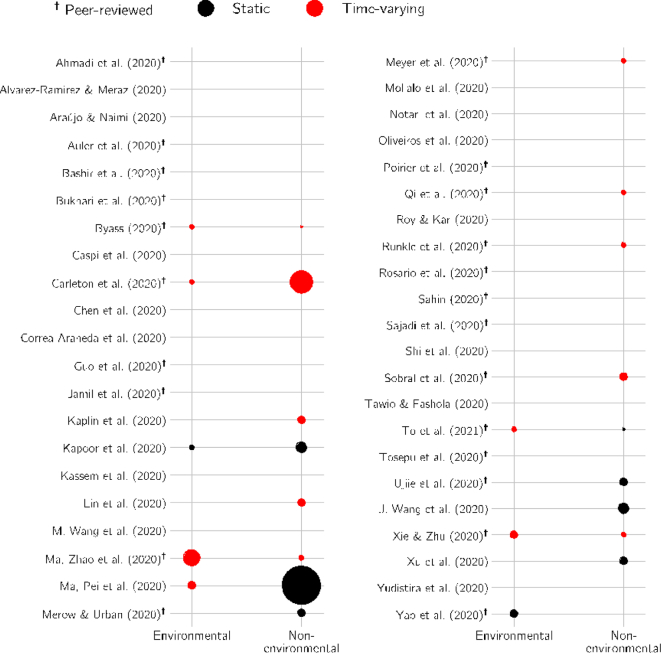
Mediating and covariate effects accounted for in individual studies. Effects are separated into environmental variables (e.g., meteorology, air quality) and non-environmental variables (e.g., demographics, mobility, non-pharmaceutical interventions, unobserved location-specific effects). The size of the scatter points is proportional to the number of effects considered.

Not accounting for the families of factors is a pervasive problem in many studies featured in this review, and less than half of all studies consider any of the aforementioned mediators and covariates (Fig. 3). Considering only the subset of global studies that accounts for mediators and covariates (compare Fig. 2, Fig. 3), however, does not reveal a consistent directionality, suggesting that detecting a potential environmental sensitivity of COVID-19 (either on seasonal or shorter timescales) is not simply a function of whether or not these effects were addressed.

### 3.1 Does China yield a clearer picture?

A comparison of findings across global studies could be complicated by a number of factors (e.g., testing capacity, non-pharmaceutical interventions, behavior and cultural practices). We next turn to studies that focus only on China. A sizable fraction of the studies included in this review concentrate on China, given its status as the original epicenter of the COVID-19 pandemic.

**Fig. 4:**
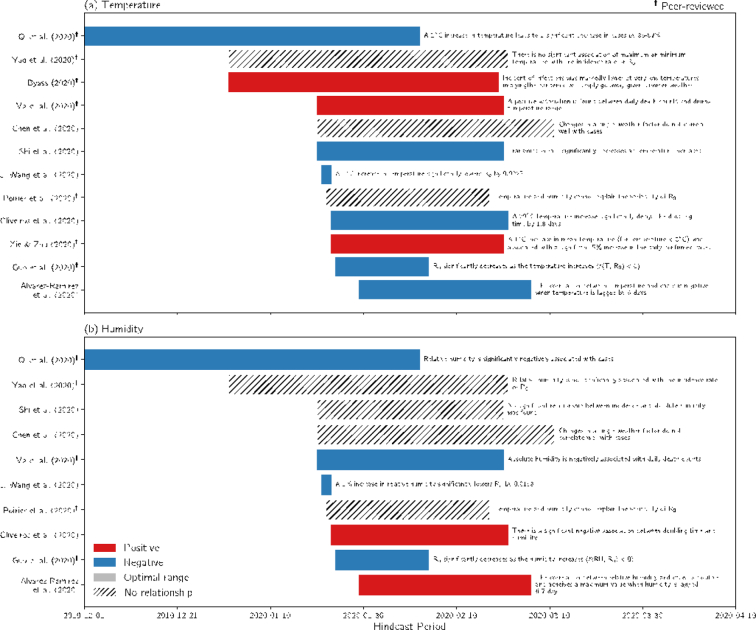
Same as Fig. 2 but for studies examining only China.

The role of temperature on COVID-19 in China exhibits even more heterogeneity than global studies (compare Fig. 4a and 2a). We detect no readily-observable trend in the sign of the temperature-COVID-19 relationship with the start of the hindcast period or length of the study. Of the 10 China-centric studies focused on the role of humidity, outcomes are relatively split among a negative relationship, a positive relationship, and no relationship (Fig. 4b).

We next consider three studies examining the effect of temperature in China: Poirier et al. [19], Oliveiros et al. [27], and Xie and Zhu [28] (Fig. 4). On the surface, these studies are very similar: they all examine the time period spanning late January to early March, use case data from the same source, and conduct their studies at similar spatial scales. Yet, these studies reach widely different conclusions (Fig. 4). Comparing the findings of these three studies (and others) is complicated by their vastly different study designs. The modeling approach in these three studies includes a generalized additive model [28], exponential and linear models [27], and Loess regression [19]. Moreover, these three studies use different dependent variables (i.e., cases, reproductive number, doubling time). As we will explain in 4.1, 4.5, each response variable and modeling approach has a host of intrinsic and extrinsic limitations, and identifying and addressing these limitations is essential when comparing results across studies.

In short, the effect of temperature and humidity, the two meteorological variables with epidemiological precedence for explaining seasonal variations in respiratory virus transmission, on COVID-19 remains unclear. Although not explored here for brevity, the effect of other variables (Fig. 1b) is similarly inconsistent. Our results are supported by recent reviews [[10], [11], [12]] as well as a recent statement by the 10.13039/501100004811World Meteorological Organization stating that “the SARS-CoV-2 virus and the COVID-19 disease do not show a robust and consistent response to temperature, humidity, wind, solar radiation, or other proposed meteorological and environmental drivers” [29].

## 4 Key methodological issues

Several of the studies detailed in Section 3 discuss the transition to boreal summer as a panacea to COVID-19 control. This idea is countered by the fact the transmission of COVID-19 continued unabated in many locations in the Northern Hemisphere during the summer of 2020, indicating that meteorological variables are not the dominant factors controlling transmission rates [30]. Despite this, several studies detailed in Section 3 have suggested that the spread of COVID-19 is constrained by the climate. We next describe several broad aspects of study designs that may invalidate results or complicate comparisons across studies.

### 4.1 Response variables

Studies considered in this review rely on a host of different dependent variables to assess environmental sensitivity. New or cumulative cases are most commonly used [e.g., 13,15,21,22,28,[31], [32], [33], [34], [35]], while others have estimated reproductive numbers [e.g., 19,[36], [37], [38], [39], [40]]. A smaller subset of the studies examined herein have calculated various rates (i.e., replication rates, infection rate, growth rate) [e.g., [41], [42], [43]]. This mix of dependent variables stands in contrast to literature on seasonal and pandemic influenza and seasonal coronaviruses, which relies heavily on the basic reproductive number [5,6] or cases [8,44].

At the pandemic's current stage, efforts to characterize the environmental sensitivity of COVID-19 rely on surveillance data, but these data are heterogeneous and impacted by biases in the time series of deaths, cases, and recoveries [12,45,46]. Even for a fixed location with quasi-homogeneous interventions, human behavior, and demographics, there are regular changes in testing policies, which could result in a different proportion of cases detected with time [47]. Similar to case counts, the basic and effective reproductive numbers (R_0_ and R_e_ or R_t_, respectively) may be affected by population characteristics and mobility and depend on time-varying susceptibility [6,12], but if biases in surveillance are systematic, R_0_ and R_e_ provide more reliable alternatives for raw case counts.

### 4.2 Mediators and covariates

The key findings of studies featured in this review are prone to interference by intervening variables. Only 18 of the 43 studies included in this review account for these effects (Fig. 3). Of the studies that do adjust, there are several additional mediating and covariate effects omitted from their study designs. Excluding non-environmental, time-varying variables which could be correlated in time with meteorological factors (e.g., non-pharmaceutical intervention implementation) may produce biased estimates. Thus, there are several pertinent questions and concerns regarding the key findings of studies that have failed to account for these important mediating and covariate effects in their study designs.

Meteorological factors influence processes relevant to virus transmission, but their impact, especially at this stage of the pandemic, is likely secondary to human contact and outbreak response measures [48,49]. Early research alludes to this: despite doubling time correlating positively with temperature and inversely with humidity, meteorological variations can only explain 18% of the overall variation in the doubling time of COVID-19 [27]. Uncovering meteorological effects on COVID-19 will likely require rigorously adjusting for other sources of variability beyond meteorological variability.

### 4.3 Pandemic phase

Data from other seasonal coronaviruses suggest that the rapid propagation of an emergent pathogen through the population during the initial phase of a pandemic is not impacted by climatic influences, exhibits no seasonality, and is driven by lack of immunity (i.e., high susceptibility) [20,44,50]. Neher et al. [44] posited that COVID-19 could transition to an endemic seasonal virus in the mid 2020s, and Engelbrecht and Scholes [20] conclude that if roughly half the population becomes infected, then meteorology-driven transmission may develop several months after introduction into the population.

Empirical studies of meteorological sensitivity are, at this stage, inherently limited by the short data record and several utilize differences in climate from different locations as proxies for seasonal variations at a fixed location [e.g., 42,51]. While a number of studies have reported empirical evidence of sensitivity [e.g., 28,34,35,[51], [52], [53], [54]], these studies rely on data collected from early community-level outbreaks, which are often inconsistent or non-representative, and do not adequately adjust for several environmental and non-environmental mediating and covariate effects.

### 4.4 Scale and extent of analysis

The studies featured in this review apply meteorological data at a wide range of spatial and temporal resolutions. Some examine fine-scale meteorological data, either from dense observational networks or global renalyses [e.g., 18,43,39]. Others, however, rely on country-averaged values [21,35], temperature in the most affected city in each country averaged over the entire period of study [16], or temperature from countries' or states' capitals [42,55]. The spatiotemporal mismatch in these and other studies is not unique to current efforts to understand the environmental sensitivity of COVID-19 but is a perennial problem in research on infectious diseases and climate [56,57].

In addition to problems of scale, studies have faced challenges in defining the appropriate range of climate zones to include in a single analysis framework. The seasonality of respiratory and influenza-like illnesses exhibits fundamental differences in temperate versus tropical regions. The seasonal cycle of influenza is characterized by a peak during winter in temperate regions, concurrent with low absolute humidity (ambient and indoor) and low temperature [4,5,57]. In the tropics, respiratory illnesses either remain high year-round or have two distinct peaks, linked to variations in precipitation [3,7]. However, the notion of temperature and humidity explaining the spread of SARS-CoV-2 in tropical regions has permeated studies featured in this review, despite weak evidence from past respiratory viruses.

Uncovering the environmental sensitivity of COVID-19 partially hinges on the reliability of the meteorological information and on the consistent availability of these data across countries and climate zones. To address this current need in the community, we have created a publicly available unified COVID-19 dataset that integrates meteorological variables with COVID-19 metrics at all administrative levels (e.g., countries, provinces/states, regions, districts) [58]. The meteorological data in this dataset are derived from the ERA5 global reanalysis and boasts higher resolution than predecessor products [59]. Similarly, Carleton et al. [60] have assembled a global, spatially resolved dataset of COVID-19 cases, location-specific containment policies, and testing regimes across 173 countries.

### 4.5 Modeling approach

Diverse modeling approaches are taken in the studies examined in this review, with the overwhelming majority of studies making use of statistical models rather than epidemiological models. Statistical models include traditional linear regression, generalized linear models, generalized additive models, and machine learning methods. The two studies that apply epidemiological models [36,61] follow the prototypical compartmental Susceptible-Infectious-Removed (SIR) model. We also note statistical models are generally trained on all available data, and we are only aware of a few studies that partition their datasets into training versus testing data [21,40,41], thus making the results applicable to prediction. While there is value both in modeling for inference and in modeling for prediction, this distinction has not always been made clear in the communication of research results to the public.

Temperature, humidity, or other meteorological variables may be spuriously correlated with COVID-19 cases (or other related metrics) by virtue of the pandemic's timing during the transition from late winter to early spring and summer of 2020. Accounting for this mutual seasonality is a necessity to distinguish causal relationships from spurious relationships. Many early studies have not addressed this source of interference, but others have accounted for the influence of trends in variables using, for example, country- or state-level time trends [e.g., 60,62].

The time-varying environmental drivers and confounders for a range of infectious diseases may have non-linear associations with the chosen dependent variable [63], and traditional linear models are insufficient to account for the complexities and evolution of an infectious disease [10]. Yet, only a few of the COVID-19 studies allow for non-linearity in their approaches [16,33,61].

Aligning surveillance data and meteorological data presents a challenge, as these two datasets are lagged by an unknown amount of time due to the incubation period of COVID-19 and recording delays. Recent work by Lauer et al. [64] found a median incubation of approximately 5 days for COVID-19, however, they point out that contemporaneous studies differ in their estimated incubation periods and have a range of approximately 2 to 14 days. The amount of time required to administer, process, and record tests is an additional source of uncertainty for determining lag and likely varies not only across geopolitical units but also in time, especially if clinics and testing centers are overwhelmed by demand for testing.

We find no consistent treatment of lag in our studies. Several studies do not consider time lags and use same-day weather and incidence data [14,16,35]. Other studies, though, test various lags ranging from −21 to 0 days [[65], [66], [67]] or use more involved approaches to determine appropriate lags (e.g., Monte-Carlo simulations to generate probability distributions for the detection delay) [40].

A positive aspect of the diversity of approaches applied to COVID-19 studies is that it is possible to assess whether results of a single study are robust to the different methods applied by complementary studies. The resulting diversity of results, however, has contributed to confusion regarding the state of understanding of climate influence on COVID-19.

## 5 Conclusions and recommendations

Despite significant statistical associations between other respiratory viruses and meteorological variables and plausible mechanisms to explain the associations, we found no consensus on how meteorology modulates the transmission of COVID-19 in 43 recent studies (Fig. 2, Fig. 4). The spatial and temporal scale of analysis, modeling approach, consideration of mediating effects and covariates, and response variables widely varied among studies. As an example, we showed that even studies with the same scale of analysis and similar data sources obtain different key results, not just in the strength of the COVID-19-meteorology sensitivity but in the direction of the relationship (Section 3.1, Fig. 4), which highlights the importance of study design as well as the complexities in uncovering a signal.

A true environmental sensitivity of COVID-19 may exist, but its impact has likely been minimal thus far in the pandemic, as compared with the influences of non-pharmaceutical interventions and human behavior [[48], [49]]. If studies claiming to have found a sensitivity of COVID-19 to meteorological factors are presented to policymakers and the public without adequate scientific vetting or without appropriate context, dissemination of such results presents potentially dangerous and even lethal misinformation and can erode scientific credibility [9].

There will undoubtedly be future efforts to assess the meteorological effects on COVID-19 as well as media attention as the Northern Hemisphere transitions into peak influenza season during winter 2020–21. To enhance the quality and robustness of future studies, we recommend the following:

### 5.1 Response variables

We suggest use of R_0_ and R_e_ as dependent variables in future studies, as these rates are less influenced by non-climatic factors compared with case counts [12]. Less-common metrics within the epidemiological community (e.g., growth rate ratios) [68] should also be explored for their efficacy in COVID-19-related studies. It may also be beneficial to determine how identical meteorological data and modeling approaches with different response variables might yield different conclusions [10]. Dependent variables used in preprint and published studies are often unclear on their intrinsic limitations and underlying biases, and, at the minimum, future studies must acknowledge how their choice of a dependent variable(s) may impact results.

### 5.2 Adjustment variables

Demographics, mobility, and non-pharmaceutical interventions all are potential effect modifiers or covariates and should be adjusted for within study frameworks. Although fully characterizing differences in testing capacity and case reporting may be impossible, attention should be given to develop proxies or at least partially adjust for these factors (e.g., correcting testing capacity based on positivity rate).

### 5.3 Pandemic phase

A clear, phase-locked seasonality may appear well before the mid 2020s, as was suggested by Neher et al. [44]. We believe it worthwhile to investigate possible environmental sensitivities during the pandemic's current stage, but caution must be taken when interpreting results that indicate an environmental sensitivity due to the limited data and mediating and covariate effects (Section 4.2). Results obtained in this first year of the pandemic could be quite different from results in subsequent years.

### 5.4 Scale and extent of analysis

Meteorological data should be matched with epidemiological data at an appropriate scale. Comparing COVID-19 cases at a national scale to a nationally averaged or single point meteorological record is inappropriate for large or climatically diverse countries. While it can be challenging to obtain sub-national COVID-19 case data in many countries, some effort should be taken to adjust data records to account for climatic heterogeneity within a country. Regarding extent, there is a critical need to understand COVID-19 dynamics, including potential climate sensitivities, in the Global South. As the relationship between climate and respiratory infections can be expected to be quite different in tropical versus temperate environments, any purported meteorological sensitivity found using a spatial or temporal subset of meteorological and surveillance data should test whether findings hold outside the given region to ensure that findings are not an artifact of the data subset or modeling approach. Moreover, future studies that examine the connections between COVID-19 and environmental variables over wide latitudinal or climatic gradients should be structured in a way that allows for different meteorological sensitivities to be identified in different climate zones, especially in temperate versus tropical regions.

### 5.5 Modeling approach

While linear statistical models are useful for exploratory data analysis, future work should focus on statistical models that can allow for non-linearities in the relationship between exposure and outcome [e.g., 63,69] or transmission-dynamic models. Accounting for the mutual seasonality of the spread of the pandemic and meteorological variables is warranted, and future studies can test whether the inclusion of time trends indicates that the influence of temperature, humidity, or other meteorological variables on COVID-19 is robust. Additionally, it is not appropriate to use same day meteorological and surveillance data, as several studies have done, and lag must be incorporated into response variables in future studies. To reduce this confusion and contribute to efficient scientific dialog through publication of results, authors could emphasize the rationale for their choice of modeling approach and explicitly state model-based assumptions and limitations. This applies to the selection of model type, the way in which interactions and nonlinearities are considered, the choice of lag length, and other key analysis details. The potential for overfitting, underfitting, and other modeling errors exists and should be addressed through cross-validation or other approaches.

## Declaration of Competing Interest

The authors declare no competing interests.
